# Status Report on COVID-19 Vaccines Development

**DOI:** 10.1007/s11908-021-00752-3

**Published:** 2021-04-14

**Authors:** Arun Kumar, William E. Dowling, Raúl Gómez Román, Amol Chaudhari, Celine Gurry, Tung Thanh Le, Stig Tollefson, Carolyn E Clark, Valentina Bernasconi, Paul A Kristiansen

**Affiliations:** Vaccine Research and Development, Coalition for Epidemic Preparedness Innovation (CEPI), Marcus Thranes Gate, 0473 Oslo, Norway

**Keywords:** SARS-CoV-2, COVID-19 vaccines, Animal models, Preclinical and clinical development, Assays and standards, SARS-CoV-2 variants

## Abstract

**Purpose of Review:**

The emergence of the severe acute respiratory syndrome coronavirus-2 (SARS-CoV-2) has affected lives of billions of individuals, globally. There is an urgent need to develop interventions including vaccines to control the ongoing pandemic.

**Recent Findings:**

Development of tools for fast-tracked testing including small and large animal models for vaccine efficacy analysis, assays for immunogenicity assessment, critical reagents, international biological standards, and data sharing allowed accelerated development of vaccines. More than 300 vaccines are under development and 9 of them are approved for emergency use in various countries, with impressive efficacy ranging from 50 to 95%. Recently, several new SARS-CoV-2 variants have emerged and are circulating globally, and preliminary findings imply that some of them may escape immune responses against previous variants and diminish efficacy of current vaccines. Most of these variants acquired new mutations in their surface protein (Spike) which is the antigen in most of the approved/under development vaccines.

**Summary:**

In this review, we summarize novel and traditional approaches for COVID-19 vaccine development including inactivated, attenuated, nucleic acid, vector and protein based. Critical assessment of humoral and cell-mediated immune responses induced by vaccines has shown comparative immunogenicity profiles of various vaccines in clinical phases. Recent reports confirmed that some currently available vaccines provide partial to complete protection against emerging SARS-CoV-2 variants. If more mutated variants emerge, current vaccines might need to be updated accordingly either by developing vaccines matching the circulating strain or designing multivalent vaccines to extend the breadth.

## Introduction

In December 2019, the Chinese health authorities reported cases of severe pneumonia in the city of Wuhan, China [1]. The severe acute respiratory syndrome coronavirus-2 (SARS-CoV-2) was identified as the causative agent accountable for the coronavirus disease 2019 (COVID-19) [1]. On March 11, 2020, the World Health Organization (WHO) declared the ongoing outbreak as a pandemic because of the severity and high potential of global spread. As of February 4, 2021, WHO reported 103 million confirmed cases and 2,260,259 deaths globally [2]. Ongoing SARS-CoV-2 infections have not only devastated human lives but also significantly damaged the financial health of both developing and developed countries. Therefore, there is an urgent need to control the pandemic by accelerating the development and mass production of efficacious vaccines against SARS-CoV-2. There has been an unprecedented effort with more than 300 vaccine candidates in various stages of development [3]; among these, more than 60 candidates have already started testing in human clinical trials [4]. These vaccines are based on both traditional and next generation platform technologies [4].

In the present review, we provide the current status of major SARS-CoV-2 vaccine development programs.

## Preclinical and Clinical Development Updates

### Animal Models for Pre-clinical Efficacy

SARS-CoV-2 infects several laboratory species including ferrets, hamsters, and non-human primates [5••]. Viral infection produces mild to moderate disease and few clinical signs, with the exception of hamsters, which develop more severe disease. The major endpoints assessed include levels of viral genomic (g)RNA or subgenomic (sg)RNA from nasal swabs, throat swabs, and bronchioalveolar lavage (BAL); presence of virus or viral RNA in tissues; lung pathology; weight loss; and imaging of lungs by X-ray, CT, or PET/CT [5••]. Common laboratory mice strains are resistant to infection, but this can be overcome through the use of adapted viruses or by use of mice that express the human angiotensin-converting enzyme 2 (ACE2) receptor through various methods. A recent article by Munoz-Fontela et al. extensively reviewed COVID-19 animal models [5••]. For vaccine and therapeutic efficacy assessments, NHP models have been widely utilized, but due to their lower cost, availability, and more severe disease phenotype, the hamster and ACE2 mouse models are now being used in many studies as well.

### Messenger RNA-based Vaccines

Messenger RNA (mRNA)-based vaccines are a relatively novel technology which, prior to the current pandemic, remained to be proven [6]. Vaccine development programs for COVID-19 are based on self-amplifying and non-amplifying mRNA formats. Alphavirus genomes have been exploited for the self-amplifying mRNA vaccines where genetic coding regions for structural proteins were replaced with gene(s) of interest [6]. Non-amplifying mRNA vaccines are of small size (2–3 kb) and encode only gene(s) of interest, regulatory elements, poly(A) tail, and a cap [7•]. The delivery of mRNA into the cytoplasm is critical; therefore, several approaches have been used including lipid nanoparticles (LNPs), cationic nano-emulsions (CNEs), and polyplexes [8]. There are 23 vaccine candidates in preclinical development, while 7 are in clinical development and 2 of them are approved for emergency use [3, 4, 9].

#### Pre-clinical Development

The mRNA-1273 (Moderna, Inc.), BNT162 (Pfizer/BioNTech), and CVnCoV (CureVac AG) vaccines protected infected macaques or hamsters from lung pathology or viral replication in lungs, but viral RNA was detected at varying levels in nasal swabs [10–13]. Three other RNA vaccines protected mice from SARS-CoV-2 challenge [10, 14, 15].

#### Clinical Development

Interim analysis from ongoing phase 2/3 trials of BNT162b2 and phase 3 trials of mRNA-1273 showed efficacy of 95% and 94.1% in two-dose schedules of 3 or 4 weeks apart, respectively [16, 17], in preventing symptomatic COVID-19. Earlier the phase 1 trials of mRNA-1273 and BNT162b1 [18, 19] showed these candidates to be safe.

Results from a phase 1/2 trial of CVnCoV vaccine [20] also showed an acceptable safety profile with dose-dependent rises in reactogenicity (local as well as systemic) and the vaccine is currently being evaluated in a phase 2b/3 clinical trial [4]. As of February 4, 2021, the remaining 4 candidates are in phase 1 and/or 2 trials (Table 1). All the vaccine candidates are being tested among adults 18 years and above except BNT162b2, which is being tested among ≥ 16-year-olds.

**Table 1:** COVID-19 vaccine clinical development pipeline by vaccine platform, as of 4th of February 2021

Platform	Phase I	Phase I/II	Phase II	Phase II/III	Phase III
RNA	Providence Therapeutics Walvax Biotechnology	Arcturus Imperial College		Pfizer; BioNTech	CureVac Moderna Therapeutics**
DNA	Symvivo OncoSec	AnGes/U. Osaka Genexine		Inovio Pharmaceuticals	Zydus Cadila
Viral vector	Beijing Wantai/U.HK IDT Immunity Merck/Themis* Merck/IAVI* ReiThera Srl Shenzhen GMI Vaxart	Cellid IIBR Shenzhen GMI			AstraZeneca** CanSino** Gamaleya Res. Institute** J&J—Janssen
Protein-based	Adimmune Covaxx Clover Biopharmaceuticals City of Hope Medicago The Finlay Vaccine Institute U. Queensland* Vaxine	Biological E CIBG—Mambisa CIBG—Abdala Nanogen Sanofi Pasteur/GSK SpyBiotech The Finlay Vaccine Institute UMN/Shionogy	Medigen Sichuan University		Anhui Zhifei Longcom FBRI SRC VB VECTOR Novavax
Live-attenuated	Codagenix				
Inactivated	U. Erciyes Shifapharmed	IMS, CAMS Valneva	Shenzhen Kangtai		Bharat** CNBG-WIBP** CNBG-BIBP** Sinovac Biotech** RIBSP**

Only include vaccine candidates started with dosing first subject

*Project is on-hold/discontinued

**Emergency use/conditional marketing approval

#### Regulatory Update

Two vaccine candidates based on mRNA platform technology, BNT162b2 and mRNA-1273, received emergency use/conditional marketing authorizations in the USA, Europe, and many other countries [9, 21].

### DNA-based Vaccines

DNA vaccines are based on a genetic engineering approach in which genes encoding target antigens are transferred into host cells with the expectation that in vivo transcription and expression of the antigen will induce immune responses and thereby protect the host. Several methods have been developed to increase DNA plasmid uptake such as gene guns (gold or wolfram DNA-coated beads) to be “shot” into the skin, jet injection with high pressure air to the skin, and electroporation, using shifting electrical pulses to drive the DNA plasmid into muscle cells or cells in the epithelial layer [22]. There are 6 vaccine developers that have started testing in humans (Table 1) and more than 16 candidates in preclinical development [4].

#### Pre-clinical

Macaques vaccinated with INO-4800 (Inovio Pharmaceuticals) had lower viral loads in lungs, but peak sgRNA in nasal swabs was high, indicating significant viral replication in the upper respiratory tract [23]. Rhesus macaques vaccinated with the GX-19 vaccine (Genexine, Inc.) showed similar results [24] as did hamsters vaccinated with another DNA vaccine delivered by jet injection [25]. Multiple Spike DNA vaccine constructs from Janssen and Harvard University demonstrated a range of protection in macaques and showed that neutralizing antibody titer significantly correlated with protection [26].

#### Clinical Development

Of the 6 DNA-based vaccine candidates in clinical testing (Table 1), INO-4800 was shown to be safe and immunogenic in a phase 1 trial (*n* = 39) when administered in two doses via intradermal (i.d.) injection followed by electroporation [27]. A phase 3 trial is on partial clinical hold pending data related to the use of the vaccine delivery device [28]. Another i.d. candidate ZyCov-D (Zydus Cadila) has entered into a phase 3 efficacy trial with a 3-dose schedule [4].

#### Regulatory Update

None of the DNA candidates have been submitted for regulatory approval based on information in the public domain.

### Viral-vectored Vaccines

Viral vectors have been exploited to deliver genes encoding antigenic proteins into host cells [29•]. Viruses with little pre-existing immunity in the target population are often selected to use as vectors, but even viruses that form the basis for widely used licensed vaccines, such as the measles virus, can be used as vectors to insert the antigen against which one wants to elicit an immune response. There are at least 15 and 40 vaccines in clinical and pre-clinical development, respectively (Table 1).

#### Pre-clinical Development

The AZD1222 (Oxford/AstraZeneca) vaccine was immunogenic in multiple species [30, 31] and protected rhesus macaques from disease, but there was a large amount of sgRNA in the upper respiratory tract [30]. The Ad26.COV2.S (Johnson & Johnson) vaccine was tested in rhesus macaques and demonstrated protection with a single dose, including no virus in the respiratory tract, no sgRNA in the BAL, 5/6 animals with no sgRNA in nasal swabs, and neutralizing antibody correlating with protection [32]. A chimpanzee Ad36 vector-based vaccine, ChAd-SARS-CoV-2-S administered intranasally led to protection in hamsters and sterilizing immunity in mice [33, 34].

The Ad5-nCoV (CanSino Biologics) vaccine protected mice and ferrets from viral challenge after intramuscular (i.m.) or intranasal (i.n.) immunization [35]. Both routes were protective, but when administered i.n, there was no virus or viral RNA detected in the upper respiratory tract in either species [35]. A different Ad5 vaccine from the Guangzhou Institutes of Biomedicine and Health protected rhesus macaques [36]. Three other Ad5 vaccines in development were immunogenic in mice [37–39], with one of them also protecting rhesus macaques from challenge [40]. Other viral-vectored vaccines including replicating vesicular stomatitis virus (VSV), lentivirus, and Newcastle disease virus (NDV)-based vaccines were immunogenic and showed protection in mice and hamsters [41–45]. Additionally, reports on modified vaccinia Ankara (MVA)-vectored vaccines also demonstrated efficacy in small animals [46–49].

#### Clinical Development

The Sputnik V (Gamaleya Research Institute) vaccine has two different adenoviral-vectored formulations (Ad26/Ad5) given as two doses 21 days apart [50]. Peer-reviewed results from phase 1/2 and phase 3 studies of this vaccine in Russia showed the vaccine to be safe, to induce cellular and humoral immune responses, and to be 91.6% efficacious [50, 51]. The Johnson & Johnson Ad26.COV2.S has shown 72% efficacy in a phase 3 study in the USA [52]. A single-dose, non-replicating Ad5-nCoV vaccine in phase 1 (*n* = 108) and phase 2 (*n* = 603) trials showed acceptable reactogenicity and tolerability as well as cellular and humoral immune response following single i.m. injection [53, 54]. Two phase 3 trials of this candidate vaccine are currently ongoing in Russia (*n* = 500) and Canada (*n* = 40,000) (Table 1). An interim analysis of the AZD1222 vaccine data from the two UK trials (COV001: phase 1/2 and COV002: phase 2/3), Brazil (COV003: phase 3), and South Africa (COV005: phase 1/2) showed a vaccine efficacy of 62.1% when given as a two-dose regimen using the same standard dose [55]. However, the efficacy increased to 90% when a low dose was followed by a standard dose in a subset of the population [55]. This was due to an inadvertent error in the dose calculation.

#### Regulatory Update

The Sputnik V was licensed for limited use by the Russian government following completion of only phase 1 trials. The vaccine has been approved for emergency use in several countries, including Argentina, the Palestinian territories, Hungary, Iran, UAE (United Arab Emirates), Mexico, and Venezuela [9].

The Ad5-nCoV was approved for limited use only in China and recently approved in Mexico and Pakistan for emergency use [9]. The data of Ad26.COV2.S has been submitted to the United States Food and Drug Administration (US FDA) for emergency use authorization (EUA) [56].

The AZD1222 vaccine has been approved under emergency use authorization by the European Medicines Agency (EMA) and other countries including the United Kingdom (UK) and India (with name Covishield), based on these data sets [9, 55], as of February 4, 2021.

### Protein-based Vaccines

Sub-unit vaccines are basically protein vaccines, where one has selected one or more immunogenic proteins or segments thereof from the pathogen to induce an immune response. The proteins can be expressed in various protein expression systems such as *E. coli*, yeast, mammalian, or insect cells and harvested and refolded in the correct three-dimensional structure to trigger the immune system. By using advanced protein engineering technology, the expressed recombinant antigenic proteins can be combined together to form nanoparticles, and thus to increase the immunogenicity. There are at least 20 vaccines in clinical and more than 100 in preclinical development [3, 4].

#### Pre-clinical Development

NVX-CoV2373 (Novavax Inc.), a nanoparticle vaccine with Matrix M adjuvant, was immunogenic in mice, baboons, and macaques [57]. The vaccinated macaques had no sgRNA in the BAL or nasal swabs and little or no inflammation in the lungs [58]. The SCB-2019 (Clover Biopharmaceuticals) vaccine was formulated with either ASO3 or CpG + alum adjuvants, both of which were immunogenic in mice, rats, and macaques and protected macaques from challenge [59]. An S1-Fc vaccine from Sorrento Therapeutics was partially protective in mice [60]. A receptor binding domain (RBD) protein-based vaccine was immunogenic in mice and protected rhesus macaques from challenge [61]. Another vaccine based on the full length S protein containing 2 proline mutations and mutated furin cleavage site elicited protection in mice [62]. Other candidates were immunogenic in mice, rabbits, and cynomolgus macaques [63, 64].

#### Clinical Development

Interim results from the phase 1 part of the NVX-CoV2372 phase 1/2 first-in-human clinical trial among Australian adults (*n* = 131) showed the vaccine candidate was safe and more immunogenic than non-adjuvanted formulation [65]. The phase 2b trial in South Africa and a phase 3 trial in UK (*n* = 15,000) are currently ongoing [66]. An interim analysis of data from UK trial showed the vaccine had ~ 90% efficacy [67].

The interim analysis results from an ongoing phase 1 trial of the SCB-2019 vaccine showed that the adjuvanted formulations were safe and immunogenic with Th1 biased cell-mediated immune response [68].

#### Regulatory Update

The EMA has started rolling review of NVX-CoV2373 vaccine.

### Live-attenuated Vaccine

An attenuated vaccine is a viral or bacterial vaccine that has its virulence/pathogenicity reduced but is still capable of replicating and triggering the immune system to respond to the whole organism. Viruses and bacteria can be attenuated through passages in culture, by removal of genes important for virulence or by a more recently developed approach of synthetically modifying the codon usage, so replication is slower due to less abundant t-RNA [69]. There is only 1 vaccine in clinical phase (Table 1) while more than 10 vaccines are in preclinical development [3].

#### Pre-clinical Development

Although several entities are reportedly pursuing live-attenuated vaccine candidates, the only publicly available pre-clinical data are for a cold-adapted virus [70]. This virus was non-lethal at high doses in K18-hACE2 mice and mice vaccinated i.n. were protected from subsequent challenge with the wild-type virus [70].

#### Clinical Development

Codagenix Inc. and the Serum Institute of India are supporting a codon-deoptimized live-attenuated vaccine that is currently in a phase I trial [4].

#### Regulatory Update

No vaccine yet in regulatory process for approval.

### Inactivated Vaccines

Inactivated viral vaccines are whole virus preparations that are no longer replication competent. Standard methods of inactivation include treating the virus with β-propiolactone (BPL) and/or formaldehyde (FA). The inactivated virus vaccines presumably contain all structural viral proteins and can potentially induce a broad immune response. There are 10 vaccines in clinical and pre-clinical development (Table 1) [3].

#### Pre-clinical Development

The BPL inactivated viral vaccine preparations CoronaVac (Sinovac), BBIBP-CorV (Beijing Institute of Biological Products) and Covaxin (Bharat Biotech International Ltd [BBIL]) were immunogenic in multiple species and protected rhesus macaques from challenge [71–73]. A BPL inactivated and split vaccine preparation showed immunogenicity in mice [74]. Vaccines inactivated by gamma irradiation or by photochemical plus ultraviolet light were protective in hamsters and mice, respectively [75–77]. Enhancement of disease, a concern for inactivated vaccines, has not been observed by clinical signs or by other endpoints.

#### Clinical Development

The CoronaVac vaccine has shown efficacy between 50 and 91% in Brazil [78]. Two phase 1/2 trials among 18–59-year and > 60-year-old individuals earlier had shown it be safe [79]. Sinopharm has developed two vaccine candidates and interim analysis of the phase 1/2 first-in-human trial of these candidates showed acceptable safety and reactogenicity profiles with the majority of the vaccine recipients also developing neutralizing antibodies against SAR-CoV-2 [80, 81]. These candidates are presently being tested in phase 3 trials and interim analysis have shown 86% vaccine efficacy (VE) in UAE and 79% in China [9]. The inactivated vaccine candidate of BBIL (Covaxin) is presently undergoing phase 3 clinical trial (*n* ~ 25,000). In the phase I trial, Covaxin was found to be well tolerated with high seroconversion rates [82].

#### Regulatory Update

CoronaVac has been given conditional approval in China and for emergency use in Brazil, Chile, Turkey, Colombia, Indonesia, and Uruguay [9].

BBIBP-CorV has already been licensed for use in China, UAE, and Bahrain and approved for emergency use in many other countries [9]. Sinopharm’s vaccine CNBG-WIIBP has been given the green light only for limited use in China and UAE [9]. India also granted approval of Covaxin for emergency use only [9].

## Comparative Analysis of Immune Assays and Reagents

For the analysis of humoral immune responses, most developers are using “in-house” panels of convalescent sera but no common reference standards to enable a fair comparison of humoral immunogenicity data across vaccine trials (Table 2a). In clinical trials where in-house convalescent sera were used to compare vaccine-elicited responses, most candidates purportedly elicit neutralizing antibody responses comparable to the median responses observed among convalescent sera; however, only two developers [65, 83] report neutralizing antibody titers in the range of the upper quartile of convalescent responses.

**Table 2:**
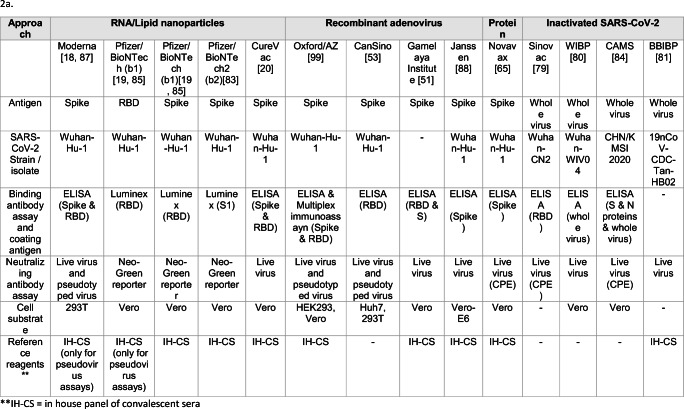

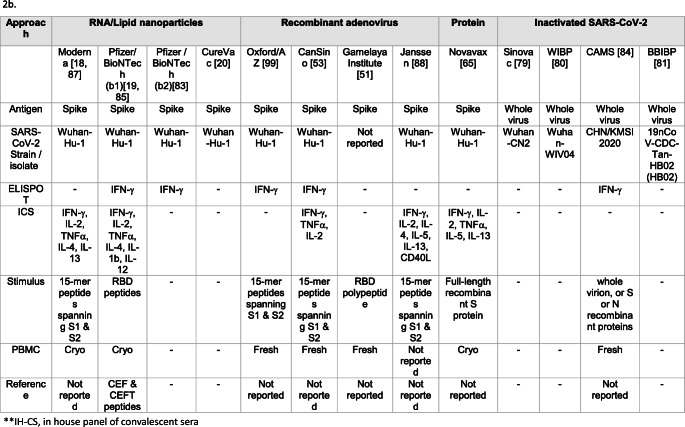
Summary of reagents, assays, and makers used in antibody (2a) and T-cell (2b) assessment among most advanced SARS-CoV-2 vaccines candidates

Table 2a offers a snapshot of the humoral immunogenicity assays, reagents, and principles across the early-stage clinical trials reported thus far. Most SARS-CoV-2 neutralizing antibody assays currently employ Vero or HEK293T cells derived either from non-human species or from kidney tissues that may not be relevant to the human respiratory epithelial cells affected in human infection. The use of cell substrates derived from human lung epithelial tissue could be explored.

There is ample evidence of induction of cell-mediated immunity (CMI) by the leading vaccine candidates licensed/approved for emergency use. Table 2b shows an overview of the various CMI assay principles, and the diversity of stimuli and experimental conditions reported to date. Most of the vaccines in advanced clinical stages have reported elicitation of IFN (interferon)-γ, assessed by either ELISPOT, and/or intracellular cytokine staining (ICS), or other assays. Other cytokines are being measured in some studies and no evidence of a predominant, vaccine-induced Th2 response has been produced thus far. Based on publicly available information, only one of the inactivated SARS-CoV-2 vaccines developed by the Chinese Academy of Medical Sciences has reported elicitation of broad CMI [84]. This is somewhat peculiar, as whole virus inactivated vaccines would be expected to elicit primarily humoral immune responses. None of the other whole virus inactivated vaccine trials has published data on induction of CMI.

The magnitude of CMI responses reported is difficult to interpret without the inclusion of reference viral peptides, such as CEF peptides (cytomegalovirus, Epstein-Barr virus, and influenza A) in CMI assays. Only one clinical trial has reported the use of such reference peptides [85]. Inclusion of such controls should be encouraged, as this has been helpful to benchmark, harmonize, interpret, and assure the quality of CMI data in clinical trials of vaccines against other pathogens.

To improve immunological assay standardization across different vaccine candidates and meaningful comparison of results, the Coalition for Epidemic Preparedness Innovation (CEPI) has supported and facilitated the development of a COVID-19 serum reference (Cat. No. 20/130) and WHO-endorsed international reference (Cat No. 20/136) available through the National Institute of Biological Standards and Controls (NIBSC). Moreover, CEPI has recently created a Centralized Laboratory Network for COVID-19 vaccine immunogenicity testing, where they have selected laboratories with high quality standards, picking the most advanced assays to be used across the network and providing all the laboratories with harmonized protocols and key reagents [86••]. The reference reagents allow comparison of data by calibration to a common reference. The Centralized Laboratory Network will allow harmonization of data through common protocols and key common reagents.

## Immune Responses Among Elderly Populations

Four leading vaccine candidates have been assessed for induction of humoral responses in the elderly [53, 83, 87, 88]. For the mRNA-1273 vaccine candidate, induction of neutralizing antibodies in the elderly was not significantly reduced in comparison to younger adults [87]. For the BNT162b1 vaccine candidate, although lower titers of neutralizing antibodies were induced in the elderly, those titers were still within the upper quartile of the titers observed in convalescent sera used as comparators [83]. Responses in the elderly induced by adenovirus vaccine candidates were more difficult to interpret, as no age stratification was reported by CanSino and only a subset of individuals over the age of 65 were included in the interim report produced by Janssen.

There are preliminary reports that in adults over 70 years of age, the mRNA-1273 vaccine was shown to elicit polyfunctional Th1 CD4 T cell responses that were comparable to those observed among vaccinees in the 18–55 age group [87]. Similarly, in a subset of adults over 65 years of age, a single dose of the Janssen Ad-26 vaccine [88] was shown to elicit bi-functional Th1 CD4 T cell responses that were either higher or comparable to those among vaccinees in the 18–55 age group. If CMI turns out to be a correlate of protection against COVID-19 severity, these preliminary observations provide hope that COVID-19 vaccines could harness CMI for the benefit of the vulnerable elderly population.

## Commonalities Across Most Advanced COVID-19 Vaccines in Clinical Phase

Commonalities begin to emerge across the most advanced vaccine candidates with late and early-stage clinical development data in the public domain. Firstly, most advanced candidates are Spike-antigen based, with the notable exception of the SARS-CoV-2 inactivated vaccines. Secondly, all Spike vaccine inserts in the leading candidates are based on SARS-CoV-2 sequences of Chinese origin, with the exception of the Sputnik V, whose Spike sequence origin was not reported in the public domain. Thirdly, all leading vaccine candidates are administered via the i.m. route, in direct contrast to the preferred non-parenteral route outlined in the WHO target product profile (TPP) [89]. Interestingly, out of 63 vaccine candidates in clinical phases only 2 of them are administered orally and 4 intranasally [4]. Lastly, aside from the CanSino and Johnson & Johnson vaccines [52, 54], which have both moved into phase III trials with a single-dose vaccine protocol, all other leading candidates have two-dose regimens. Among the other leading candidates, one protein [65] and three mRNA formulated in LNPs [18, 20, 83] vaccines have moved forward to either phase III trials or EUA with two-dose regimens, all aiming for the second immunization to be administered within a month after the first immunization. Four inactivated viral vaccines produced in China have also moved into phase III under a two-dose vaccine regimen [79–81, 84]. In summary, for most leading candidates, a two-dose regimen will be necessary, and this may again contrast with the WHO TPP expectations of a single-dose primary series and the yearly boosting doses originally anticipated for long-term use.

## SARS-CoV-2 Variants and Impact on Vaccine Development

Evidence suggests that mutations occur at a high frequency in the Spike and receptor binding motif (RBM), as a result of recombination events and other positive pressures [90] and that the RBM is the most divergent region of S [91] which may lead to the emergence of escape mutants. Variants with several S mutations have been detected in Brazil, Denmark, South Africa, the UK, and USA [92]. Variants B.1.1.7 and B.1.351 which were first reported in the UK and South Africa, respectively, have caused a great deal of concern regarding their potential impacts on vaccine efficacy. Recent data from in vitro studies demonstrate that vaccinee sera from several different vaccines have significantly decreased neutralization activity against variant B.1.351 compared to older viral isolates while the sera show little or no decreased neutralization against variant B.1.1.7 [52, 67, 93–95]. Phase III trial results from Novavax and AstraZeneca revealed that the vaccines had high efficacy against the B.1.1.7 variant (85.6% and 74.6%, respectively) [67, 96]. However, efficacy for the Novavax, Johnson & Johnson, and AstraZeneca vaccines were much lower against the B.1.351 variant (49.4%, 57% and <25%, respectively), although the percent efficacy of the AstraZeneca vaccine was only reported for mild and moderate disease [52, 67, 97]. Based on these data, there is an urgent need to start development of updated versions of leading vaccines in order to protect against the B.1.351 variant, and also to develop a framework for expanded genomic surveillance and rapid analysis of new variants to generate actionable data.

To strengthen surveillance for mutations and variants of concern, CEPI formed a partnership with the GISAID Initiative (www.gisaid.org), which hosts the largest open access pooled dataset of SARS-CoV-2 genomes (*n* = 544,478 full genomes as of 23 February 2021). The GISAID Initiative’s EpiCoVTM application (www.epicov.org) enables comparative analyses and modeling of sequences and builds a mutation risk map building on knowledge of epitopes and deep mutation scanning experiments [98].

To rapidly respond to emerging variants, CEPI set up a collaborative project named “Agility” in partnership with Public Health England (PHE), NIBSC, and the GISAID Initiative, to enable the rapid biological assessment of emerging variants both in vitro and in vivo. The Agility project aims to provide open-access high quality reports on the biological implications of emerging variants and inform the need for strain changes or adaptions for vaccines to ensure effectiveness is maintained.

## Conclusions

Vaccine development is a lengthy process normally involving a 10–15-year timeline. Collaborative efforts and access of next generation technologies, animal models, calibrated and harmonized assays and standards, and active engagement of regulatory agencies with developers allowed approval of vaccines against COVID-19 within an unprecedented 9 months after identification of the SARS-CoV-2 virus.

## Abbreviations

ACE2: Angiotensin-converting enzyme 2
BAL: Bronchioalveolar lavage
BPL: β-Propiolactone
CMI: Cell-mediated immunity
CEPI: Coalition for Epidemic Preparedness Innovation
CNEs: Cationic nano-emulsions
COVID-19: Coronavirus disease 2019
EMA: European Medicines Agency
EUA: Emergency use authorization
FA: Formaldehyde
FDA: Food and Drug Administration
gRNA: Genomic RNA
LNPs: Lipid nanoparticles
mRNA: Messenger RNA
MVA: Modified vaccinia Ankara
NIBSC: National Institute of Biological Standards and Controls
PHE: Public Health England
RBD: Receptor binding domain
RBM: Receptor binding motif
SARS-CoV-2: Severe acute respiratory syndrome coronavirus-2
sgRNA: Subgenomic RNA
TPP: Target product profile
VE: Vaccine efficacy
VSV: Vesicular stomatitis virus
WHO: World Health Organization
